# Common and Distinct Alterations of Cognitive Function and Brain Structure in Schizophrenia and Major Depressive Disorder: A Pilot Study

**DOI:** 10.3389/fpsyt.2021.705998

**Published:** 2021-07-20

**Authors:** Mengying Ma, Yuyanan Zhang, Xiao Zhang, Hao Yan, Dai Zhang, Weihua Yue

**Affiliations:** ^1^Institute of Mental Health, The Sixth Hospital, Peking University, Beijing, China; ^2^Key Laboratory of Mental Health, Ministry of Health & National Clinical Research Center for Mental Disorders, Peking University, Beijing, China; ^3^PKU-IDG/McGovern Institute for Brain Research, Peking University, Beijing, China

**Keywords:** major depressive disorder, schizophrenia, cognitive function, gray matter volume, superior frontal cortex

## Abstract

**Objective:** Numerous studies indicate that schizophrenia (SCZ) and major depressive disorder (MDD) share pathophysiological characteristics. Investigating the neurobiological features of psychiatric-affective disorders may facilitate the diagnosis of psychiatric disorders. Hence, we aimed to explore whether patients with SCZ and patients with MDD had the similar or distinct cognitive impairments and GMV alterations to further understand their underlying pathophysiological mechanisms.

**Methods:** We recruited a total of 52 MDD patients, 64 SCZ patients, and 65 healthy controls (HCs). The Measurement and Treatment Research to Improve Cognition in Schizophrenia (MATRICS) Consensus Cognitive Battery was used to assess cognitive functions. In addition, voxel-based morphometry (VBM) analysis was used to evaluate the gray matter volume (GMV) by using MRI scanning. One-way ANOVA and *post-hoc* tests were used to find the differences among the MDD, SCZ, and HCs. Finally, we explored the correlation between structural alterations and cognitive functions.

**Results:** Compared with that of HCs, processing speed was impaired in both patients with SCZ and patients with MDD (*F* = 49.505, *p* < 0.001). SCZ patients displayed impaired cognitive performance in all dimensions of cognitive functions compared with HCs (*p* < 0.001, except social cognition, *p* = 0.043, Bonferroni corrected). Whole-brain VBM analysis showed that both SCZ and MDD groups had reductions of GMV in the medial superior frontal cortex (cluster-level FWE *p* < 0.05). Patients with SCZ exhibited declining GMV in the anterior cingulate cortex and right middle frontal cortex (MFC) compared with HCs and MDD patients (cluster-level FWE *p* < 0.05). The mean values of GMV in the right MFC had a positive correlation with the attention/vigilance function in patients with MDD (*p* = 0.014, partial. *r* = 0.349, without Bonferroni correction).

**Conclusions:** In total, our study found that MDD and SCZ groups had common cognitive impairments and brain structural alterations, but the SCZ group exhibited more severe impairment than the MDD group in both fields. The above findings may provide a potential support for recognizing the convergent and divergent brain neural pathophysiological mechanisms between MDD and SCZ.

## Introduction

Approximately 1% of the population suffers from schizophrenia (SCZ), which is one of the top 10 causes of disability worldwide (1). The clinical character of SCZ consists of varying degrees of behavioral anomalies, cognitive impairment, and emotional aberrations (2). Moreover, major depressive disorder (MDD) is a common psychiatric disorder with a high disabling effect (3) and a high relapse rate (4). It is characterized by a persistently low mood accompanied by anhedonia, psychomotor retardation (5, 6) and cognitive impairment (7). SCZ has some common symptoms overlapping with the MDD (8), such as mood symptoms, social withdrawal, and cognitive deficits (9). Moreover, they also share some common genetic loci (10). Previous data have shown that the prevalence of depressive disorder in schizophrenia was around 40% (11). The above results suggest SCZ and MDD may have some common endophenotype characteristics, while each disease has a specific pathophysiological mechanism. However, it is still unclear that the neural changes of the common and specific mechanism in SCZ and MDD.

Meanwhile, as one of the core characteristics of SCZ, cognitive impairment covers almost all main dimensions (12), including mental speed, working memory, attention, executive function, etc. (13, 14). Moreover, except mood disturbances, patients with MDD usually exhibit impairments of cognitive functions (15). Meta-analyses showed that MDD patients had moderately declined cognitive functions (16, 17). Compared with healthy controls (HCs), MDD patients exhibit decreased performance in several domains of cognitive functions, including information processing speed, working memory, verbal learning, memory, visuospatial learning and memory (15, 18–20). Hence, patients with SCZ may share considerable overlaps with MDD in several dimensions of cognitive function, especially processing speed and working memory.

The reductions in GMV of the prefrontal cortex were observed consistently in SCZ (21). Moreover, the decreased GMV in prefrontal-related regions, such as the right orbitofrontal cortex and dorsolateral prefrontal cortex was also exhibited in MDD (22). Previous study has been conducted on the GMV alterations of MDD and SCZ with the finds of the decreased GMV in middle frontal cortex (MFC) and medial prefrontal cortex (MPFC) (23). We considered that the abnormal structural alterations of the frontal cortex may be the common signatures of SCZ and MDD. However, few studies have focused on the similar or distinct GMV alterations of frontal cortex in patients with SCZ and patients with MDD. Further investigation is necessary.

In addition, structural alterations in the brain may have relationships with cognitive impairments in individuals. For instance, previous studies reported that deficit of working memory was associated with the structural changes in the prefrontal cortex, superior temporal gyrus, anterior cingulate cortex, medial frontal cortex, and hippocampal subregion in patients with SCZ (24–26). Processing speed was correlated with the structural alterations in the middle frontal gyrus, inferior frontal gyrus, bilateral orbitofrontal cortex, bilateral superior temporal gyrus, and the memory function had a correlation with the decreased GMVs in bilateral orbitofrontal cortex (27–29). Meanwhile, the GMV alterations of the inferior frontal gyrus were significantly associated with sustained attention in patients with MDD (30). Acoustic and visual attention was correlated with abnormal GMVs in the thalamus and amygdala/parahippocampal gyrus in patients with MDD (31).

Taken together, the above findings suggest that several similar or distinct cognitive impairments and frontal regional structural abnormalities might exist in patients with SCZ and patients with MDD, which the cognitive functions might have associations with the structural alterations. Besides, neuroimaging techniques have been well-known and widely applied in the study of psychiatric disorders (32). Voxel-based morphometry (VBM) is a useful approach in examining the whole-brain structural alterations (33). Hence, the aim of our study was to explore the common or distinct alterations of cognitive functions and brain structural in patients with MDD and patients with SCZ, compared with HCs by using the Measurement and Treatment Research to Improve Cognition in Schizophrenia (MATRICS) Consensus Cognitive Battery (MCCB), T1-weighted structural magnetic resonance imaging (MRI), and the association between cognitive function and GMV alterations.

## Methods and Materials

### Subjects

A total of 64 SCZ patients, 52 MDD patients, and 65 HCs were recruited. Both the SCZ and the MDD patients were recruited from Peking University Institute of Mental Health. Patients were evaluated by two clinical psychiatrists using the Diagnostic and Statistical Manual of Mental Disorders, Fourth Edition, Text Revision (DSM-IV-TR) diagnostic criteria of SCZ or MDD, without any other comorbidities of the DSM-IV-TR Axis I Disorders. HCs who were recruited from the community were assessed by psychiatrists using the Structured Clinical Interview for DSM-IV-TR Axis I Disorders, Research Version, Non-Patient Edition (SCID-I/NP) (34), and subjects with any psychiatric disorders were excluded.

The inclusion criteria were (1) being from 18 to 55 years of age, (2) having Han Chinese lineage, (3) being right-handed, and (4) patients with SCZ or MDD. The exclusion criteria were (1) diagnosis with any neurological disease, (2) being unconscious more than 5 min, (3) contraindications for MRI scanning, (4) patients who underwent electroconvulsive therapy over the previous 6 months, (5) patients with a history of any severe physical diseases, (6) patients with any monogenic inherited diseases, (6) patients with serious impulsive behavior/suicide attempts, and (7) patients during pregnancy and lactation.

There were 5 SCZ patients and 14 MDD patients who were drug-naïve. In addition, 59 SCZ patients received atypical antipsychotics (such as olanzapine, risperidone, aripiprazole, amisulpride, paliperidone, and clozapine). The Haloperidol equivalent dose of the antipsychotics was 11.03 ± 6.1 mg/day (35). In addition, 38 MDD patients received serotonin reuptake inhibitors (SSRI) (escitalopram, sertraline, fluoxetine); serotonin norepinephrine reuptake inhibitors (SNRI) (venlafaxine, duloxetine); and noradrenergic and specific serotonergic antidepressants (NaSSA) (mirtazapine). The equivalent citalopram dose of the antidepressants was 26.18 ± 11.82 mg/day (36).

The present study was approved by the Ethics Committee of the Peking University Institute of Mental Health. Informed consent forms were signed by subjects themselves or their legal guardians after getting detailed information about the study.

### Cognitive Function Measurement

The Measurement and Treatment Research to Improve Cognition in Schizophrenia (MATRICS) Consensus Cognitive Battery (MCCB) was designed to comprehensively and systematically evaluate the cognitive functions in individuals and further facilitate the development of medications for the treatment of cognitive deficits (37, 38). Also, the MCCB Chinese version has good reliability and validity for the Chinese population (39). We used the MCCB to assess the cognitive functioning of patients with SCZ, patients with MDD and HCs, including processing speed, attention/vigilance, working memory, verbal learning, visual learning, reasoning and problem solving and social cognition.

### MRI Data Acquisition

All subjects were scanned using a 3.0-Tesla GE Discovery MR750 scanner at the Center for MRI Research, Peking University Institute of Mental Health. We collected the T1-weighted structural images of the whole brain of all subjects. The parameters of T1-weighted structural imaging were, using a T1-weighted fast spoiled gradient recalled (FSPGR) sequence, repetition time (TR) = 6.66 ms, echo time (TE) = 2.93 ms, field of view (FOV) = 256 × 256 mm^2^, matrix size = 256 × 256, flip angle (FA) = 12°, voxel size = 1 × 1 × 1 mm^3^, slice thickness = 1 mm, and slice gap = 0 mm. In total, it contained 192 slices of T1-weighted structural images.

### Processing and Analyses of the MRI Data

Matlab 2013b and SPM (http://www.fil.ion.ucl.ac.uk/spm) were used to analyze the imaging data. We used the the VBM approach to analyzing the structural MRI data.

### Structural Image Preprocessing

(1) We checked for artifacts of all images. (2) The origin of each image was corrected to match the anterior commissure. (3) The T1-weighted structural images were segmented into gray matter, white matter, and cerebrospinal fluid. (4) The segmented images were aligned and normalized from the original space to the Montreal Neurological Institute (MNI) space template using the DARTEL approach. (5) In order to reduce the effect of noise and compensate for the alignment error in the spatial normalization process, the images were smoothed with an 8 mm full width at half maximum (FWHM) Gaussian smoothing kernel.

### Analysis of MRI Data

After controlling for age, gender, education, and total volume of the whole brain, we used one-way ANOVA to analyze GMV among the three groups. To avoid edge effects, voxels were included only when their absolute values were >0.2 and added a gray matter mask in the analysis. We extracted mean values of GMV of each region with significant group differences from ANOVA to perform *post-hoc* analysis. The significance level was set at *p* <0.05 with whole-brain family-wise error (FWE) correction.

### Statistical Analyses of the Demographic and Cognitive Function Data

We analyzed demographic and cognitive function data by using a standard statistical package (IBM SPSS 21.0, Chicago, IL), including one-way ANOVA, chi-square tests, and *post hoc* analysis. Given the above results of VBM analyses, we extracted the mean values of GMV from the regions of altered GMV and performed the partial correlations analysis of the cognitive functions of patients, controlling for age, gender, and education. The partial correlation analysis was performed in R. The significance level was set at *p* <0.05 after Bonferroni correction for multiple comparisons.

## Results

### Demographic and Cognitive Function Results

We recruited 64 SCZ patients, 52 MDD patients, and 65 HCs. All groups had similar age distribution. In addition, there were more males with SCZ—~1.5 times as many as females—which was in line with the findings that the male/female incidence rate of SCZ was about 1.4:1 (40). There were more females with MDD, approximately twice as many as males, which was consistent with the epidemiological findings that the prevalence of depression in women was about twice that of men (41). Although the MDD patients and HCs had achieved similar educational levels, the SCZ patients had lower educational levels (Table 1). Hence, we included gender and education as covariates in subsequent analyses.

**Table 1:** Demographic and behavioral characteristics of schizophrenia patients, major depressive disorder patients, and healthy controls.

**Characteristic**	**SCZ patients (SD)**	**MDD patients (SD)**	**HCs (SD)**	***F/X^**2**^***	***p*-value**
Age (years)	26.67 (9.34)	24.98 (4.8)	25.25 (4.07)	1.55	0.317
Gender (female/male)	26/38	34/18	32/33	7.141	0.028
education (years)	13.64 (2.89)	16.04 (2.66)	16.83 (2.12)	26.685	<0.001
Speed of processing	50.36 (8.97)	59.06 (6.97)	62.68 (5.1)	49.505	<0.001
Attention/vigilance	48 (7.86)	56.4 (7.7)	58.37 (5.96)	37.208	<0.001
Working memory	47.52 (7.79)	50.17 (9.02)	53.97 (8.67)	9.442	<0.001
Verbal learning	49.95 (9.71)	60.27 (6.51)	59.05 (5.98)	33.279	<0.001
Visual learning	51.44 (9.54)	59.1 (5.46)	60.28 (5.02)	29.097	<0.001
Reasoning and problem solving	53.13 (9.64)	58.9 (7.86)	59.67 (6.62)	12.104	<0.001
Social cognition	37.17 (7.23)	40.39 (8.28)	40.62 (8.23)	3.707	0.026

*SCZ, schizophrenia; MDD, major depressive disorder; HCs, healthy controls; SD, standard deviation*.

**Figure 1:**
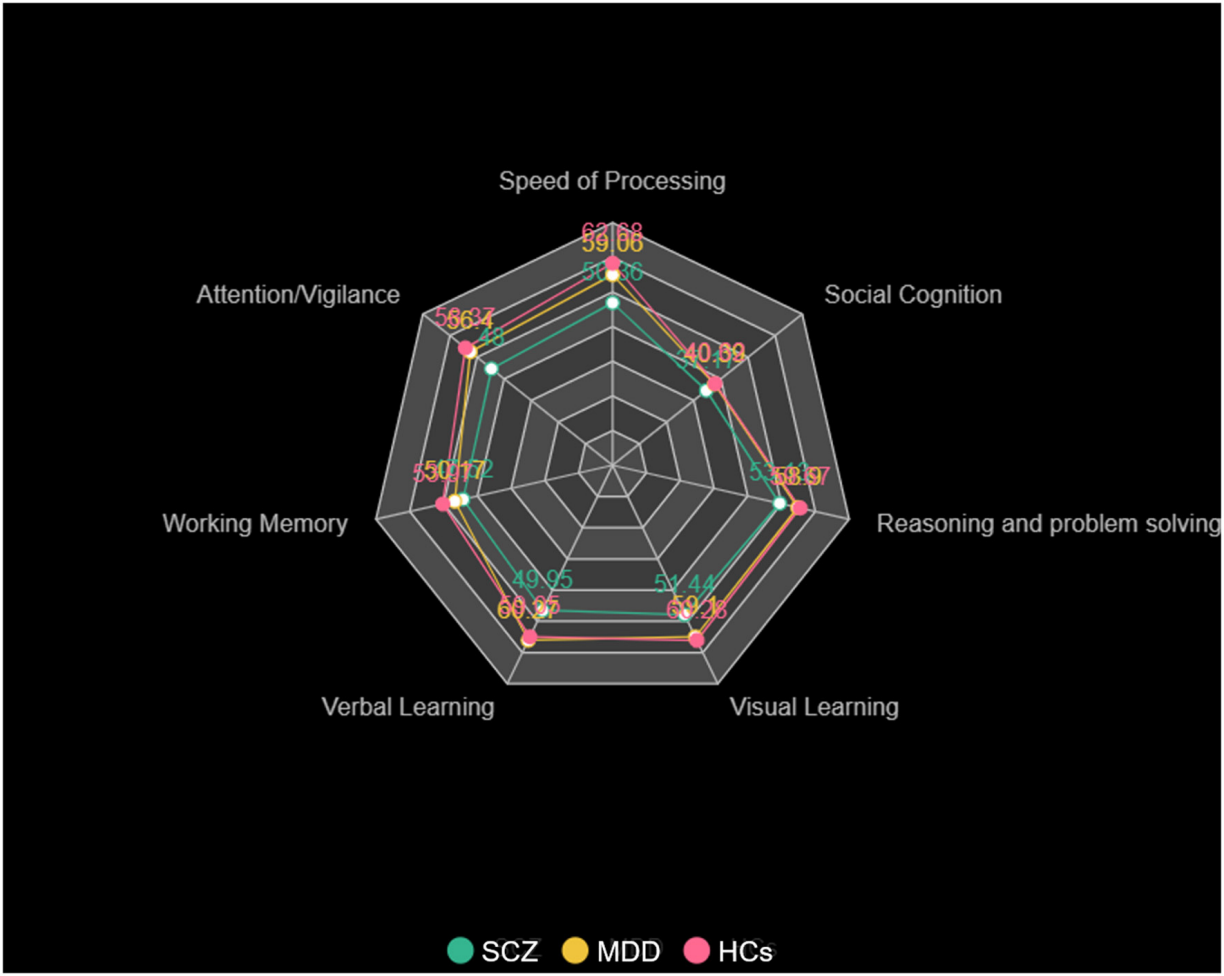
Cognitive function of patients with SCZ, patients with MDD, and HCs. The cognitive scores for processing speed, attention/vigilance, working memory, verbal learning, visual learning, reasoning, problem solving, and social cognition in patients with SCZ, patients with MDD, and HCs.

There were significant differences in cognitive functioning, including processing speed, attention/vigilance, working memory, verbal learning, visual learning, reasoning, problem solving, and social cognition among the three groups (Table 1, Figure 1). *Post-hoc* analysis showed that patients with SCZ had worse performance of the cognitive functions in the whole dimension compared with HCs (*p* < 0.001, except social cognition, *p* = 0.043, Bonferroni corrected). It was noticed that compared with HCs, MDD patients also had worse performance in the field of processing speed (*t* = −3.619, *p* = 0.023, Bonferroni corrected), except SCZ patients. Meanwhile, MDD patients showed a trend of impairment in the dimension of working memory compared with HCs (*t* = −3.796, *p* = 0.051, Bonferroni corrected).

### Gray Matter Volume Results

**Figure 2:**
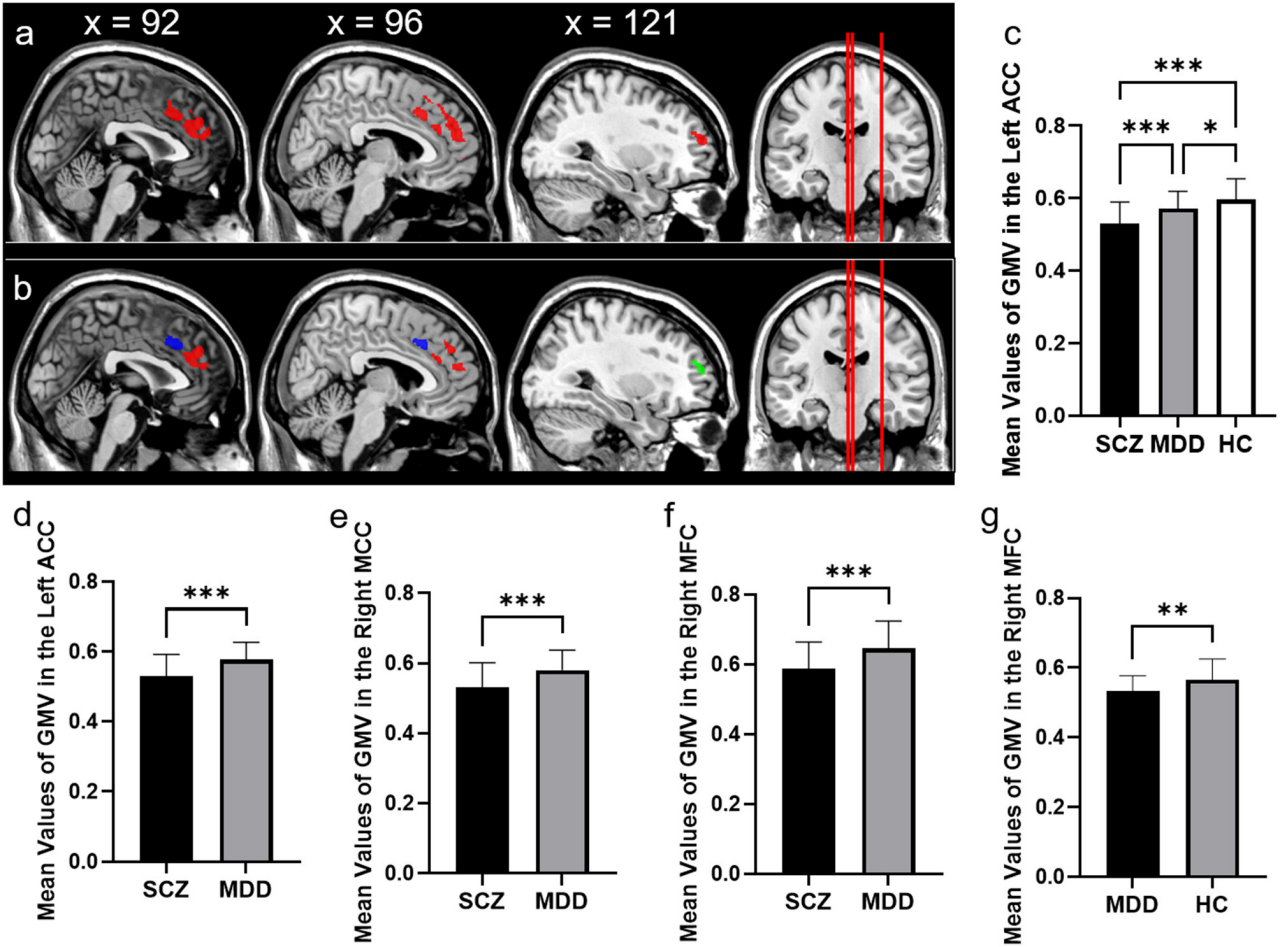
Comparison of GMV in schizophrenia, major depressive disorder, and healthy controls. **(a)** The significant brain region that showed significant differences among patients with SCZ, patients with MDD, and HCs. **(b)** The significant brain region which showed significant differences between SCZ patients and MDD patients (controlling for age, gender, education, and total volume of the whole brain, *p* < 0.05, cluster-level whole-brain FWE corrected). The bar graphs showed the mean values of GMV in the left anterior cingulate cortex among three groups. **(c)** The left anterior cingulate cortex between SCZ and MDD. **(d)** The right median cingulate cortex between SCZ and MDD. **(e)** The right middle frontal cortex between SCZ and MDD. **(f)** The right middle frontal cortex between MDD and HC. **(g)** ACC, anterior cingulate cortex; MCC, median cingulate cortex; MFC, middle frontal cortex, *p < 0.05, **p < 0.01, ***p < 0.001, Bonferroni corrected.

After controlling for age, gender, education and total volume of the whole brain, the GMV in the left anterior cingulate cortex (ACC) (*x* = 2, *y* = 36, *z* = 26, *T* = 23.97, cluster size = 2,181) showed significant difference among three groups, which involved the right medial of superior frontal cortex (MSFC) and right median cingulate cortex (MCC) (*p* < 0.05 whole-brain cluster level FWE corrected, Table 2, Figure 2). Among this large brain region, patients with SCZ showed less GMV in the left ACC (*x* = 2, *y* = 38, *z* = 21, *T* = 4.77, cluster size = 639), right MCC (*x* = 3, *y* = 15, *z* = 39, *T* = 4.29, cluster size = 283) and right middle fontal cortex (MFC, *x* = 32, *y* = 54, *z* = 15, *T* = 4.18, cluster size = 130) compared with patients with MDD. Besides, compared with HCs, MDD patients showed reduced GMV in the right MSFC (*x* = 14, *y* = 53, *z* = 20, *T* = 4.94, cluster size = 666) and SCZ patients had decreased GMV in the left ACC (*x* = 2, *y* = 36, *z* = 26, *T* = 6.92, cluster size = 2,181), which also extended to the right medial superior frontal cortex (MSFC) and right median cingulate cortex (MCC). Finally, we did not find any region in patients with MDD or patients with SCZ had significantly larger GMV compared with that in HCs.

**Table 2:** Results of GMV analysis of the patients with schizophrenia patients, major depressive disorder patients and healthy controls (controlling for age, gender, education, and total volume of the whole brain, *p* < 0.05, cluster-level whole-brain FWE corrected).

**Hemisphere**	**Brain region**	**Cluster size**	**MNI coordinates**	**Peak *F/t* value**	**Cluster-level *p*_FWE_**
			(*x, y, z*)		
**ANOVA**					
Left	Anterior cingulate cortex	2,181	2, 36, 26	23.97	<0.001
Right	Medial superior frontal cortex		2, 38, 21	22.79	
Right	Median cingulate cortex		3, 21, 39	20.19	
**MDD** **>** **SCZ**					
Left	Anterior cingulate cortex	639	2, 38, 21	4.77	0.001
Right	Median cingulate cortex	283	3, 15, 39	4.29	0.006
Right	Middle frontal cortex	130	32, 54, 15	4.18	0.021
**HC** **>** **MDD**					
Right	Medial superior frontal cortex	666	14, 53, 20	4.94	0.001
Right	Medial superior frontal cortex		5, 56, 26	4.94	
Right	Superior frontal cortex		17, 57, 5	4.76	
**HC** **>** **SCZ**					
Left	Anterior cingulate cortex	2,181	2, 36, 26	6.92	<0.001
Right	Medial superior frontal cortex		6, 56, 21	6.45	
Right	Median cingulate cortex		3, 21, 39	6.35	

### Correlation Analysis

**Figure 3:**
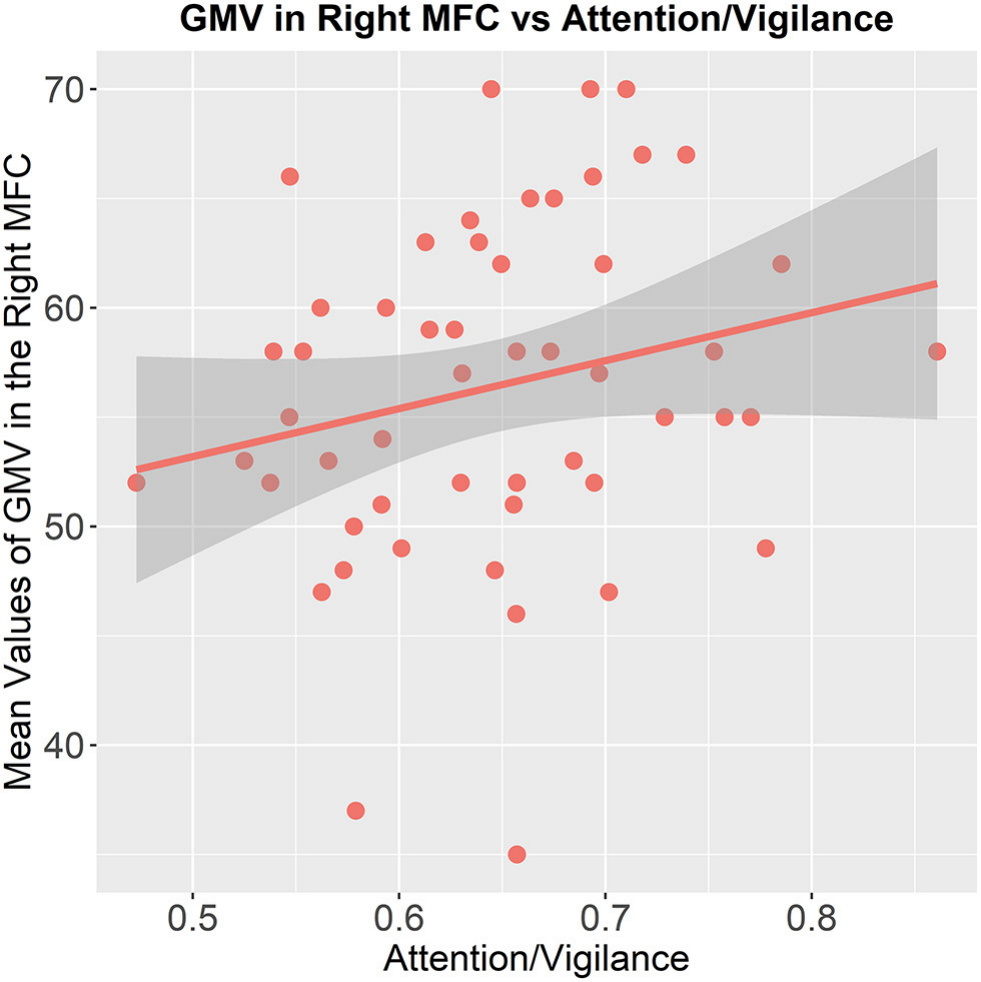
Association between the GMV of the brain region and cognitive functioning. The mean values of GMV in the right middle frontal cortex are correlated to the attention/vigilance function in patients with MDD (*p* = 0.014, partial. *r* = 0.349, controlling for age, gender, and education, without Bonferroni correction).

In the patients with MDD, we found the mean values of GMV in the right MFC (*x* = 32, *y* = 54, *z* = 15), which showed significant difference between patients with MDD and patients with SCZ had a significant positive correlation with the cognitive function of attention/ vigilance (*p* = 0.014, partial. *r* = 0.349, controlling for age, gender, and education, without Bonferroni correction, Figure 3). While we did not find the mean values of GMV in the left, ACC (*x* = 2, *y* = 36, *z* = 26) had associations with cognitive functions in SCZ patients.

## Discussion

In this study, we have analyzed the cognitive functioning and brain GMV changes in SCZ patients, MDD patients, and HCs. Our findings were the following: (1) compared with HCs, both the SCZ patients and MDD patients exhibited impaired cognitive functioning in processing speed, which suggested the impairment of the executive function was the common characteristic of the two diseases; (2) SCZ patients also showed deficits in cognitive functioning in the other dimensions, including attention/vigilance, working memory, verbal learning, visual learning, reasoning, problem solving, and social cognition compared with HCs, while MDD patients exhibited a trend impairment of working memory; (3) compared with HCs, both the patients with SCZ and the patients with MDD showed decreased GMV in the right MSFC; (4) patients with SCZ exhibited reduced GMV in the left ACC and right MFC compared with HCs and MDD patients; (5) the mean values of GMV in the right MFC had a positive correlation with the cognitive function in the attention/vigilance field in patients with MDD. The above results suggest that SCZ and MDD have common and distinct cognitive impairments and brain structural signatures. Both the SCZ patients and the MDD patients showed abnormal cognitive functioning with respect to processing speed and decreased GMV in the right MSFC. Moreover, patients with SCZ showed impaired cognitive functioning in all dimensions and less GMV in the left ACC and right MFC.

### Cognitive Function

#### Common Cognitive Impairment in Speed of Processing

The finding that both the patients with SCZ and the patients with MDD showed information processing speed deficits was in accordance with previous research (15, 42–45). Information processing speed, as a major part of executive function, plays an important role in learning and memory cognitive function (15). The impaired function of processing speed may implicate neural circuitry underlying cognitive and mood abnormalities in individuals with depression (43, 46). Besides, previous data have also reported that the worse processing speed was related to the severity of psychosis (47, 48). Moreover, impaired processing speed can successfully predict functional outcomes in patients with SCZ and may be an important predictor of the conversion to a full-blown psychiatric disorder in individuals at high risk (47, 49–51). Therefore, the deficit in speed of information processing may be an underlying shared pathophysiological mechanism of cognitive functions, mood and psychiatric symptoms impairments between SCZ and MDD.

#### Distinct Cognitive Impairments in SCZ Patients

In line with our expectation, compared with HCs and MDD patients, patients with SCZ had worse cognitive function in all dimensions, including/processing speed, attention/vigilance, working memory, verbal learning, visual learning, reasoning, problem solving, and social cognition. This result has been confirmed by previous studies. Although subjects showed impairments in memory, executive function, attention, and processing speed function among the three groups SCZ, MDD, and bipolar disorder (BD), patients with SCZ exhibited more impairment than the rest of the subjects (45). SCZ has a significant association with cognitive decline (52). Cognitive impairment such as the stable phenotypes of SCZ significantly contribute to functional abnormalities in patients with SCZ (53, 54). A meta-analysis found that the reduced brain-derived neurotrophic factor (BDNF) levels and elevated C-reactive protein (CRP) in SCZ had a significant relationship with cognitive impairment, particularly in subjects with chronic SCZ (55). Moreover, the decreased GMV in the paralimbic system in SCZ had correlations with cognitive functioning, clinical variables, and symptomatology (27). These results suggest that cognitive impairment is a stable phenotype of SCZ, which has inflammatory, neurotrophic, and structural brain foundations.

### Brain Structure

#### Common Decreased GMV in the Right MSFC

Both the SCZ group and the MDD group showed significantly reduced GMV in the right medial of superior frontal cortex, compared with that in HCs. The superior frontal cortex is generally considered a crucial brain region involving the emotional regulation and cognitive control function (56, 57). Previous studies have reported that the abnormal activity in the superior frontal cortex might be related to excessive self-referential processing and impairment in emotional cognitive control processing in patients with MDD (58). A large number of studies found that the structure of the superior frontal cortex had an important association with depressive symptoms among different populations (59–62). One study reported that the GMV alterations in superior frontal cortex was associated with the severity of depression in patients with MDD (63). Furthermore, a recent study found that the GMV of the left supplementary motor area, superior frontal cortex, and precentral gyrus had negative correlations with the hallucination severity and positively correlated with the responsive search score (64). Goghari found that compared with controls, nonpsychotic relatives of patients with SCZ exhibited less GMV in the superior and inferior frontal cortex regions, in which aspects of decreased GMV in the prefrontal cortex might reflect genetic liability for SCZ (65). Hence, the decreased GMV of the right superior frontal cortex associated with emotional regulation, cognitive control function, and psychiatric symptoms may be a potential common pathophysiological signature both of MDD and SCZ.

#### Distinct Decreased GMV of the Left ACC in Patients With SCZ

Our findings are consistent with previous reports that have shown that the reductions of GMV in parts of the prefrontal and cingulate were specifically related to SCZ (66, 67). The ACC has been reported to play a crucial role in pathophysiology of SCZ (68). A recent study suggested that the decreased perfusion in the ACC might be related to the development of delusions in SCZ (69). Besides, the reduction of GMV in the ACC has important associations with both of negative symptoms and positive symptoms in SCZ (66, 70). Meanwhile, the ACC is also involved in the integration of sensory stimuli, which suggests that the abnormal structure in ACC may disturb the integration of sensory stimuli and contribute to delusions and grandiosity thought disorder in patients with SCZ (70). Findings from previous proton magnetic resonance spectroscopy (MRS) research have shown that abnormal ACC glutamate and gamma aminobutyric acid (GABA) levels has been observed across the illness course, in antipsychotic-treated and drug-naive/off-medication patients with SCZ (71, 72). Thus, the decreased GMV of the right ACC may be a potential biomarker for the diagnosis and treatment of SCZ.

#### The Reduction of GMV in the Right MFC Is Correlated With the Attention/Vigilance Function

A study has shown that the ventromedial frontal cortex had direct influence on attention function by the connections with higher-order sensory regions (73). In addition, the ventromedial frontal cortex can also influence selective attention processes underlying visual search through communicating with ventral visual regions (46, 74). Moreover, the integrity and coordinated function of medial PFC indeed plays an important role in the cognitive function of attention (75). A previous study reported that the dorsolateral prefrontal cortex (including the caudal MFC), supplementary motor area, and posterior cingulate cortex participated in the dorsal attention network, which would be active during attention-demanding tasks (76). Therefore, though narrowly escaping statistical significance, the decreased GMV in the right MFC may be crucial for the attention/vigilance function in MDD patients.

There are several limitations that should be acknowledged in the present study. First, the sample size is slightly small. As a pilot study, we would like to recruit more subjects to test and verify the current results. Second, the present findings may be limited by the study's cross-sectional design. Moreover, we did not distinguish between the patients treated with drugs and those without drug treatment, so the results need to be treated with caution. In the future, we would like to follow up with patients and distinguish patients treated with drugs from those without drug -treatment to further explore changes in cognitive functioning and brain structure.

Collectively, our study indicated that both abnormality of the cognitive impairment in information processing speed and reductions in GMV in the right medial superior frontal cortex were related to emotional regulation, executive control function, and psychiatric symptoms, and these may be the common pathophysiological foundations for both diseases. Besides, cognitive impairment may be the stable phenotype for patients with SCZ, and the decreased GMV of the right ACC may be a potential biomarker for the diagnosis of SCZ. The above results may provide several clues for further exploration of the diagnosis and treatment of SCZ and MDD.

## Data Availability Statement

The original contributions presented in the study are included in the article/supplementary material, further inquiries can be directed to the corresponding authors.

## Ethics Statement

The studies involving human participants were reviewed and approved by the Ethical Committee of the Peking University Sixth Hospital. The patients/participants provided their written informed consent to participate in this study.

## Author Contributions

WY and DZ designed and supervised the study. MM and YZ recruited subjects performed the study and organized the data. MM analyzed the data and wrote the paper. XZ, HY, and WY gave instruction for the analysis and modified the paper. All authors contributed to the article and approved the submitted version.

## Conflict of Interest

The authors declare that the research was conducted in the absence of any commercial or financial relationships that could be construed as a potential conflict of interest.
